# Autophagy in Retinal Ganglion Cells in a Rhesus Monkey Chronic Hypertensive Glaucoma Model 

**DOI:** 10.1371/journal.pone.0077100

**Published:** 2013-10-15

**Authors:** Shuifeng Deng, Mei Wang, Zhichao Yan, Zhen Tian, Hongrui Chen, Xuejiao Yang, Yehong Zhuo

**Affiliations:** 1 State Key Laboratory of Ophthalmology, Zhongshan Ophthalmic Center, Sun Yat-sen University, Guangzhou, People’s Republic of China; 2 Sun Yat-Sen Memorial Hospital, Sun Yat-sen University, Guangzhou, People’s Republic of China; University of Rochester, United States of America

## Abstract

Primary open angle glaucoma (POAG) is a neurodegenerative disease characterized by physiological intraocular hypertension that causes damage to the retinal ganglion cells (RGCs). In the past, RGC damage in POAG was suggested to have been attributed to RGC apoptosis. However, in the present study, we applied a model closer to human POAG through the use of a chronic hypertensive glaucoma model in rhesus monkeys to investigate whether another mode of progressive cell death, autophagy, was activated in the glaucomatous retinas. First, in the glaucomatous retinas, the levels of LC3B-II, LC3B-II/LC3B-I and Beclin 1 increased as demonstrated by Western blot analyses, whereas early or initial autophagic vacuoles (AVi) and late or degraded autophagic vacuoles (AVd) accumulated in the ganglion cell layer (GCL) and in the inner plexiform layer (IPL) as determined by transmission electron microscopy (TEM) analysis. Second, lysosome activity and autophagosome-lysosomal fusion increased in the RGCs of the glaucomatous retinas, as demonstrated by Western blotting against lysosome associated membrane protein-1 (LAMP1) and double labeling against LC3B and LAMP1. Third, apoptosis was activated in the glaucomatous eyes with increased levels of caspase-3 and cleaved caspase-3 and an increased number of TUNEL-positive RGCs. Our results suggested that autophagy was activated in RGCs in the chronic hypertensive glaucoma model of rhesus monkeys and that autophagy may have potential as a new target for intervention in glaucoma treatment.

## Introduction

Glaucoma is a neurodegenerative disease characterized by physiological intraocular hypertension and the progressive death of retinal ganglion cells (RGCs) [1,2]. However, the cellular and molecular pathophysiology of glaucoma is poorly understood. Experimental and clinical evidence has shown that progressive RGC loss can be attributed to apoptosis [1,3], whereas the blockade of axonal transport, as induced by the intraocular pressure (IOP) elevation, is considered one of the most important mechanisms regulating neuronal apoptosis [1]. Intervention via apoptosis regulation has been indicated as a leading approach to protect neurons in glaucoma [4]. Several recent studies have reported that autophagy is also activated in RGCs and plays a role in autophagic cell death after IOP elevation [5–8].

Autophagy, another mode of programmed cell death, is a dynamic, multi-step process that is also termed autophagic flux [9,10]. A natural autophagic process starts when a flat membrane system wraps around a portion of the cytosol and/or organelles, forming a closed double-membrane bound structure called an autophagosome. The autophagosome then fuses with lysosomal vesicles to degrade the cargo contents, forming an autolysosome [9–11]. Through this stepwise maturation process, autophagy delivers long-lived proteins or unwanted organelles into the lysosome for degradation and reuses the resulting amino acids, thus maintaining the quality control of essential cellular components as well as of cellular homeostasis [11]. Morphologically, the autophagic vacuoles (AVs) can be classified into early or initial AVs (AVi), which are typically referred to autophagosomes, and late or degraded AVs (AVd), which are typically referred to autolysosomes [9]. AVi are fused with lysosomes to form AVd; thus, the inhibition of AVi-lysosome fusion due to lysosomal defects can be detected as an accumulation of AVi [9,12]. In contrast, as fusion occurs, enzymes and membrane proteins are delivered to the AVd from the lysosome; thus, the AVi do not contain the lysosomal membrane proteins or enzymes, whereas the AVd do contain these elements [13,14]. Deficiencies in lysosomal membrane proteins, i.e., LAMPs, can disturb autophagosome maturation, thus resulting in increased amounts of AVi or both AVi and AVd [9,15]. This observed relationship indicates that an estimation of the formation of AVi and AVd and the lysosomal membrane proteins approximates whether the autophagic flux is increased or reduced. 

Autophagy is important in maintaining the cellular homeostasis of the nervous system, as malfunctions in autophagy within the central nervous system (CNS) can reduce the lifespan or induce neurodegenerative diseases in rodent models [16–19]. Autophagy alterations have also been observed in many human neurodegenerative diseases. Increased autophagic flux or blocked autophagic flux has been observed in brain neurons after acute insults such as cerebral ischemia and traumatic brain injury and after chronic insults such as Parkinson’s disease and Alzheimer’s disease [12,19–22]. Autophagy has also been studied in glaucoma neurodegeneration over the last five years. Seok Hwan Kim [5] and N Rodrı´guez-Muela [6] suggested that autophagy may be activated in RGCs after optic nerve transection, as the expression levels increase of several autophagy-related genes and autophagy marker proteins. Antonio Piras [7] demonstrated that autophagy is activated in RGCs in an acute high-intraocular-pressure rat model, as autophagy marker protein expression levels increased; however, the lysosomal activity did not concurrently decrease. Recently, H-Y Lo Pilly Park [8] suggested that autophagy is activated in RGCs in a chronic hypertensive glaucoma rat model, which was demonstrated in vivo by an increased expression of autophagy marker proteins together with the accumulation of AVs in RGCs by TEM analysis. However, AV accumulation and the increased expression of autophagy marker proteins may be a result of the blockage of autophagic degradation instead of increased autophagy flux [9,15]. Therefore, a simple determination of the total number of AVs and of autophagy marker protein expression levels is insufficient for an overall estimation of autophagic activity in vivo. Quantification of the relative numbers of AVi and AVd observed by TEM and the estimation of fusion events between AVi and lysosomes can help discriminate between autophagy activation and blocked autophagosome maturation [23]. Thus, in the present work, we used different methods to estimate the entire autophagy process in a rhesus monkey model of chronic hypertensive glaucoma, investigating not only AV abundance but also lysosomal activity and autophagosomal fusion.

We found that autophagy, in addition to apoptosis, was activated in a chronic hypertensive glaucoma model in rhesus monkeys. This result may predict a potential therapeutic prospect for chronic hypertensive glaucoma by intervening in autophagic regulation rather than in apoptosis regulation. To our knowledge, autophagy in RGCs has not been previously studied in a rhesus monkey model of chronic glaucoma. 

## Materials and Methods

### Ethics Statement

This study strictly adhered to the ARVO Statement for the Use of Animals in Ophthalmic and Vision Research and was approved and monitored by the Institutional Animal Care and Use Committee of Zhongshan Ophthalmic Center (Permit Number: SYXK (YUE) 2010-0058). The rhesus monkeys used in this study were from the Ophthalmic Animal Laboratory, Zhongshan Ophthalmic Center, Sun Yat-sen University. The animals were housed in an air-conditioned room with an ambient temperature of 16–26°C, a relative humidity of 40–70% and a 12-hour light-dark cycle with a daytime light intensity of approximately 200 lux. The animals were individually housed in stainless steel wire-bottomed cages with sufficient space (800 mm wide, 800 mm depth and 1600 mm height) and provided with a commercial primate diet. In addition to normal pellet food, fresh fruit was provided twice daily, and water was freely available at all times. Animal health was monitored daily by the animal care staff and veterinary personnel. Additional enrichment and welfare were provided; for example, we routinely introduced toys into the home cage environment (often containing enjoyable food items) and played music in the room. Furthermore, we spent half an hour interacting with the monkeys directly before the experiment; we believe that this interaction with humans helped reduce any potential stress related to the experiment. All surgery was performed under anesthesia with an intramuscular injection of ketamine hydrochloride (5 mg/kg, Ketalar 50®, GuTian Pharmaceuticals Ltd, Fujian, China) plus chlorpromazine hydrochloride (2.5 mg/kg, Ketalar 50®, GuTian Pharmaceuticals Ltd, Fujian, China), and topical proparacaine HCl (Alcaine®, 0.5%, Alcon Laboratories, Ft. Worth, TX) was used for all procedures involving contact with the cornea. All efforts were made to minimize suffering.

### Animals and ocular examinations

Four adult rhesus monkeys (three males and one female, six to seven years old, weighing 5-8 kg) were used in this study. All monkey eyes underwent ocular examinations pre- and post-laser treatment including IOP measurements, fundus photographs and quantitative assessments of peripapillary retinal nerve fiber layer (RNFL) thickness. The IOP was measured with a Tono-penXL tonometer (Reichert, Depew, NY); IOP measurements were performed three times in each eye after surface anesthesia, and the measurements were averaged. The IOP of each eye was monitored for one week (once per day) pre-laser treatment and weekly after laser photocoagulation. The optic disc was detected through fundus photographs using a retinal camera (TRC-50DX RETINAL CAMERA; Topcon, Tokyo, Japan). RNFL thickness was quantitatively examined by optic coherence tomography (OCT; STRATUS OCT, Carl Zeiss Meditec, Dublin, CA, USA). Fundus photographs and OCT examinations were performed after pupil dilation with 2.5% phenylepherine HCl (Mydfrin, Alcon) and 1% tropicamide HCl (Mydriacil, Alcon); two examinations were performed once every two weeks after intraocular hypertension was achieved. 

OCT (STRATUS OCT; Carl Zeiss Meditec, Dublin, CA, USA) was employed to quantitatively measure the peripapillary RNFL thickness. Three 3.4-mm diameter circular scans, centered on the optic disk, were obtained for each eye. Individual scans were assessed for quality (good focus, adequate signal-to-noise ratio and well-centered circular scan around the optic disk) [24]. The data from the three acceptable quality scans were averaged and analyzed.

### Establishment of a chronic hypertensive glaucoma model in rhesus monkeys

After the monkeys were anesthetized, a green diode laser and slit lamp delivery system (VISULAS Trion; Carl Zeiss Meditec AG, Goeschwitzer Strasse, Jena, Germany) was used to produce contiguous burns over 360° of the angular circumference of the mid-trabecular meshwork (TM) [25] in one eye that was selected at random (140–170 spots; 50 μm in diameter; 0.5 s duration; 1,200 mW in power); the other eye served as a control. Tobramycin dexamethasone eye drops (Mydriacil, Alcon) were applied to the surgical eye immediately after the laser treatment to relieve the inflammatory reaction. Intraocular hypertension was defined as a rise in IOP above 21 mmHg. Subsequent laser session(s) were required if no IOP elevation occurred within one month or if the elevated IOP decreased to a normal level for more than one month. The animals were sacrificed when the cup/disc ratio extended to equal or more than 0.7 with a continuous decrease in the RNFL thickness, which demonstrated glaucomatous RGC damage in the laser-treated eye [26]. Then, we obtained samples for examination with TEM, Western blotting and immunohistochemistry assays.

### Tissue preparation

**Figure 1:**
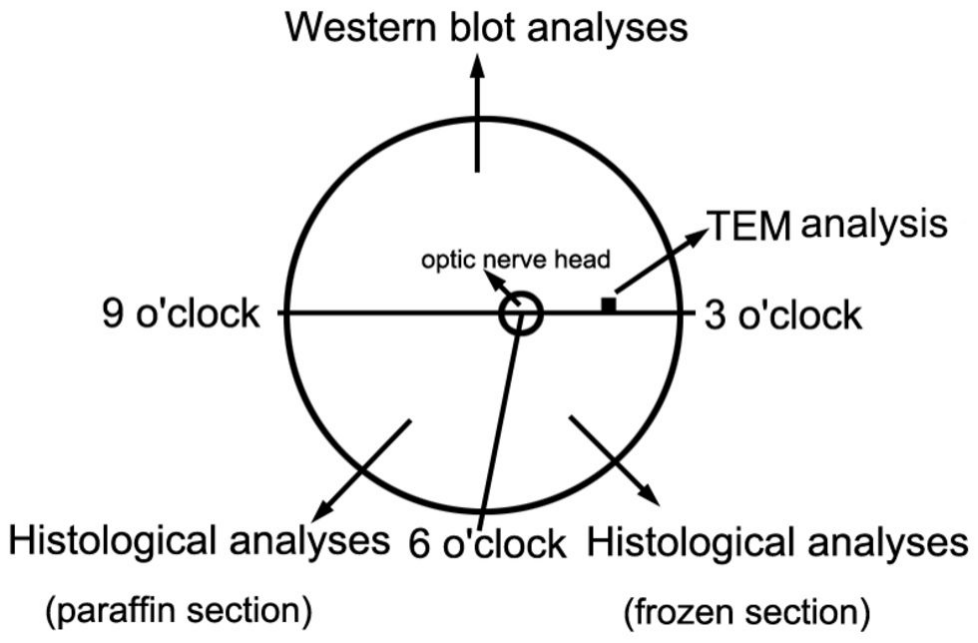
Tissue preparation for each retina.

The animals were deeply anesthetized with an intramuscular injection of ketamine HCl (15 mg/kg) plus chlorpromazine hydrochloride (10 mg/kg) and then perfused transcardially with 2 L of phosphate-buffered saline (PBS) solution. Each eye was quickly enucleated and then sectioned in half from the 3 to 9 o'clock positions, which were marked before the cardiac perfusion; thus, the superior and inferior sections of the eyeball were obtained. Under a light microscope (Zeiss), in the coincident position of the superior retinal sections of each eye (along the section line and approximately 4 mm away from the nasal rim of optic nerve head), a 1 × 1 mm retinal section was removed and post-fixed with 4% glutaraldehyde in 0.1 mM/L cacodylate buffer (pH 7.4) for TEM examination (Figure 1). The remaining retina of the superior section was isolated for Western blot analyses (Figure 1). The inferior section was dissected into two sections crossing the direction of the optic nerve head and the 6 o'clock position. Then, the nasal section was placed in fixative (4% paraformaldehyde in 0.1 M PB, pH 7.4) for paraffin sectioning and immunoperoxidase or immunofluorescence analyses, whereas the bitemporal section was embedded in an optimal cutting temperature (OCT) compound (Tissue-Tek, Sakura Finetek USA, Torrance, CA) and stored at -80°C for frozen sectioning and immunofluorescence analyses (Figure 1).

### TEM analysis

After the retinal sections were post-fixed overnight at 4°C, vibratome sections (100 µm) were cut, rinsed in cacodylate buffer, post-fixed in osmium tetroxide (1% in cacodylate buffer) for 30 min and contrasted in uranyl acetate (1% in ethanol 70%) for 5 min. The sections were dehydrated in graded alcohol and embedded in Durcarpan ACM. Ultrathin sections (0.1 mm) were cut, mounted on formvar-coated slot grids, contrasted with uranyl acetate and alkaline lead citrate and visualized with TEM (Zeiss Inc., Thornwood, NY, USA). In randomly selected fields, we captured 25 non-repeating micrographs (at 9,700×) of the three layers in the retinal section including the RNFL, GCL and IPL. The area of one such micrograph was then regarded as the unit area. The numbers of AVi and AVd per unit area in each layer were counted and analyzed using the SAS system. Close double-membrane-bound vacuoles containing morphologically intact cytoplasm or organelles were identified as AVi, whereas double- and multiple-membrane vacuoles containing partially disintegrated or electron-dense contents or whorls of membranous material were identified as AVd [9].

### Western blot analyses

After being washed twice in the PBS solution, the retinal extracts of both control and injured eyes were homogenized in RIPA buffer. Tissue extracts were incubated for 30 min on ice and ultrasonicated for 15 min before being clarified by centrifugation at 12,000×g for 30 min at 4°C. The supernatants were assayed for protein content using the Bio-Rad DC Protein Assay Kit (Bio-Rad laboratories, Milan, Italy) after measuring the protein concentrations of the sample using a standard BCA assay (Key Gen Bio TECH, Nanjing, China). An equal amount of total protein was separated by SDS polyacrylamide gel electrophoresis (12% for LC3B, cleaved caspase-3 and caspase-3; 10% for LAMP1 and Beclin 1) and transferred to polyvinylidene difluoride (PVDF) membranes (Bio-Rad, Hercules, CA). After blocking with 5% BSA for 1 h at room temperature (RT), the primary antibodies were incubated overnight at 4°C followed by a species-specific HRP-conjugated secondary antibody for 1 h at RT. The protein bands were visualized by incubation with chemiluminescence substrates (ECL Plus; Perkin Elmer Inc., Covina, CA). The blots were developed and analyzed with Image J software, and all assays were performed on at least three separate experiments. The protein levels were normalized to GAPDH or β-actin.

The primary antibodies and dilutions used were as follows: anti-Beclin 1 (#3495, Cell Signaling, Boston, MA, USA) 1:1,000; anti-LC3B (#3868, Cell Signaling, Boston, MA, USA) 1:1,000; anti-GAPGH (#2118, Cell Signaling, Boston, MA, USA) 1:1,000; anti-cleaved caspase-3 (#9661, Cell Signaling, Boston, MA, USA) 1:500; anti-caspase-3 (sc-7272, Santa Cruz, CA, USA) 1:200; anti-LAMP1 (ab25630, Abcam, HK, China) 1:1,000; and anti-β-actin (Mab1445, Multiscience, Hangzhou, China) 1:1,000. The HRP-conjugated goat anti-mouse or goat anti-rabbit secondary antibodies (Multiscience, Hangzhou, China) were used at 1:10,000. 

### Histological analyses

Immunohistochemistry was performed on 6-μm cryostat sections or paraffin sections. The cryostat sections were cut, mounted onto superfrost ultra plus glass slides (Adhesion Microscope Slides Guangzhou, China) and fixed with acetone for 15 min, whereas the dewaxing paraffin sections were pre-treated using heat-mediated antigen retrieval with a sodium citrate buffer (pH 6) for 15 min and then immersed for 20 min in 0.3% H_2_O_2_ in methanol to quench endogenous peroxidases. 

For immunoperoxidase labeling, the pre-treated paraffin sections were rinsed in PBS and preincubated for 45 minutes with a 5% BSA solution containing 0.3% Triton. The sections were then incubated overnight with primary antibody diluted in 2% BSA solution containing 0.1% Triton X-100, washed in PBS and incubated with the biotinylated secondary antibody (Multiscience, Hangzhou, China) for 2 h at RT. After three PBS washes, the sections were incubated in an avidin–biotin–peroxidase complex (ABC Reagent; Multiscience, Hangzhou, China) for 2 h at RT and then developed by incubation with diaminobenzidene (Multiscience, Hangzhou, China) substrate solution until the desired staining intensity was obtained. Finally, the sections were dehydrated in graded alcohol and mounted in Eukitt for examination by light microscope (Zeiss). 

For immunofluorescence labeling, the sections were preincubated for 45 min with 5% BSA solution containing 0.3% Triton X-100 and then incubated overnight at 4°C with the primary antibody (anti-LC3B: 1:200, #3868, Cell Signaling, Boston, MA, USA; anti-LAMP 1:1:50, ab25630, Abcam, HK, China) in 2% BSA solution containing 0.1% Triton X-100. Then, the sections were washed in PBS and incubated for 2 h at RT with fluorochrome-coupled secondary antibody (Alexa Fluor 488 or Alexa Fluor 555 from Multiscience, Hangzhou, China). The sections were then rinsed in PBS, mounted using VECTARSHIELD mounting medium with DAPI (Beyotime, Guangzhou, China) for 5 min, washed, cover-slipped and mounted in anti-fade fluorescence mounting medium (Applygen Technologies, Beijing, China) before examination using a confocal laser scanning microscope (Zeiss). We used a LSM 510 Meta confocal microscope (Carl Zeiss Jena GmbH, Jena, Germany). For double labeling, immunoreactive signals were sequentially visualized in the same section with two distinct filters, with acquisition performed in a separate mode. The images were processed with LSM 510 software and mounted using Adobe Photoshop.

### TUNEL staining

Using the In Situ Cell Death Detection Kit (Roche Applied Science, Mannheim), 10-mm cryostat sections were used for TUNEL staining following the manufacturer’s protocol and then examined by confocal laser scanning microscopy (Zeiss).

### Statistical analyses

A paired design was applied in this study. Data from pre- and post-laser-treated eyes were compared using paired *t*-tests. Thus, the data on IOP and RNFL thickness were evaluated using paired *t*-tests. The data from the Western blot were evaluated using a Wilcoxon matched-pairs signed-ranks test, as the difference in the data between the experimental eyes and normal eyes was outside of the normal distribution. The data for TEM analysis were evaluated using a linear mix model analysis. All assays were performed at least in triplicate. All statistical assessments were two-sided, and *P*<0.05 was considered significant. The data are presented as the mean ± standard deviation (SD). All analyses were performed using SAS 9.1 software.

## Results

### Glaucoma damage of RGC after chronic IOP elevation

**Figure 2:**
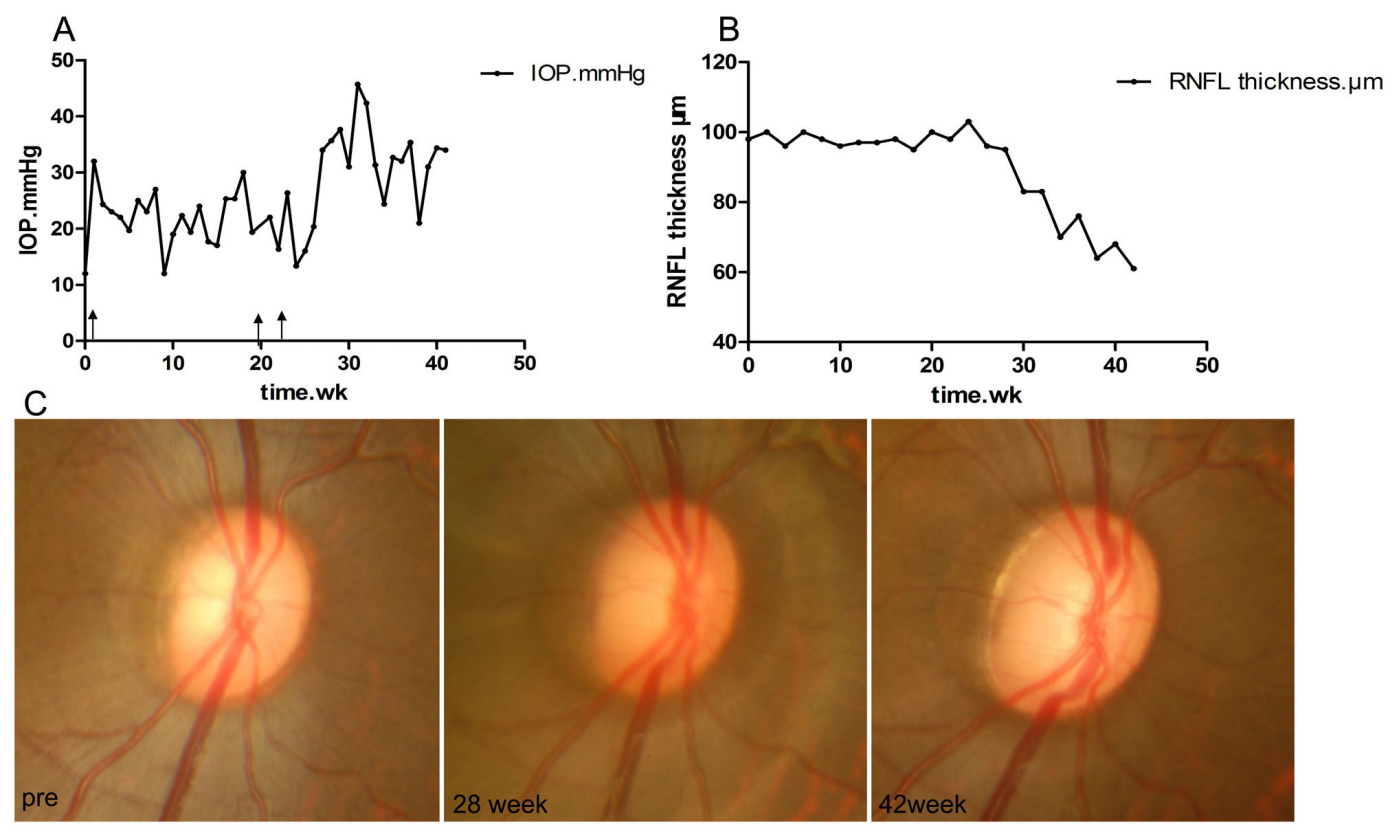
The chronic experimental glaucoma (exp gl) damage. A. The IOP elevation process after laser photocoagulation of the right eye of one monkey. The solid arrows indicate the laser treatments. B. The damage process to the RNFL thickness. Corresponding to the sustained elevated IOP in A, the RNFL thickness attenuated gradually after 28 weeks. C. Progressive optic disc cupping, corresponding to A.

Each eye had a baseline IOP below 21 mmHg, and no ocular abnormalities were observed pre-laser treatment in the fundus photographs or on the RNFL thickness quantitative examinations (Figure 2; Table 1). After one to three laser photocoagulation treatments, the IOP of the four treated eyes gradually elevated, accompanying a progressive optic disc cupping. Finally, the average IOP of the laser-treated eyes ranged from 24.9±6.8 to 52.9±8.2 mmHg (mean ± SD), which was significantly (*P*<0.05) higher than that of the control eyes, and the IOP elevation duration ranged from 14 to 49 weeks (Figure 2; Table 1). Concurrently, the cup/disc ratio in laser-treated eyes lengthened to 0.7~0.8, whereas it was approximately 0.3 pre-laser treatment (Figure 2; Table 1). Furthermore, the average RNFL thickness of the laser-treated eyes was accordingly continuously attenuated (Figure 2; Table 1). The glaucomatous RGC damage was indicated by the enlargement of the cup/disc ratio and by the attenuation of RNFL thickness.

**Table 1:** Summary of Study Animals.

Number	Mean IOP (mmHg)* # (pre-treated/post-treated)	Peak IOP (mmHg)	Duration (wk)	Cup/Disc Ratio (initial/final)	RNFL thickness (μm) *(initial/final)
M1	16.0±1.4/24.9±6.8	38.0	36	0.3/0.8	115/79
M2	12.0±1.7/25.3±8.1	45.7	48	0.3/0.7	98/61
M3	17.3±1.4/34.2±10.2	56.3	49	0.3/0.8	91/73
M4	15.7±1.5/52.9±8.2	64.3	14	0.3/0.8	100/41

^*^ *P*<0.05.

^#^ Values are the mean ± SD.

### Expression of Beclin 1, LC3B-II and LC3B-II/LC3B-I after chronic IOP elevation

**Figure 3:**
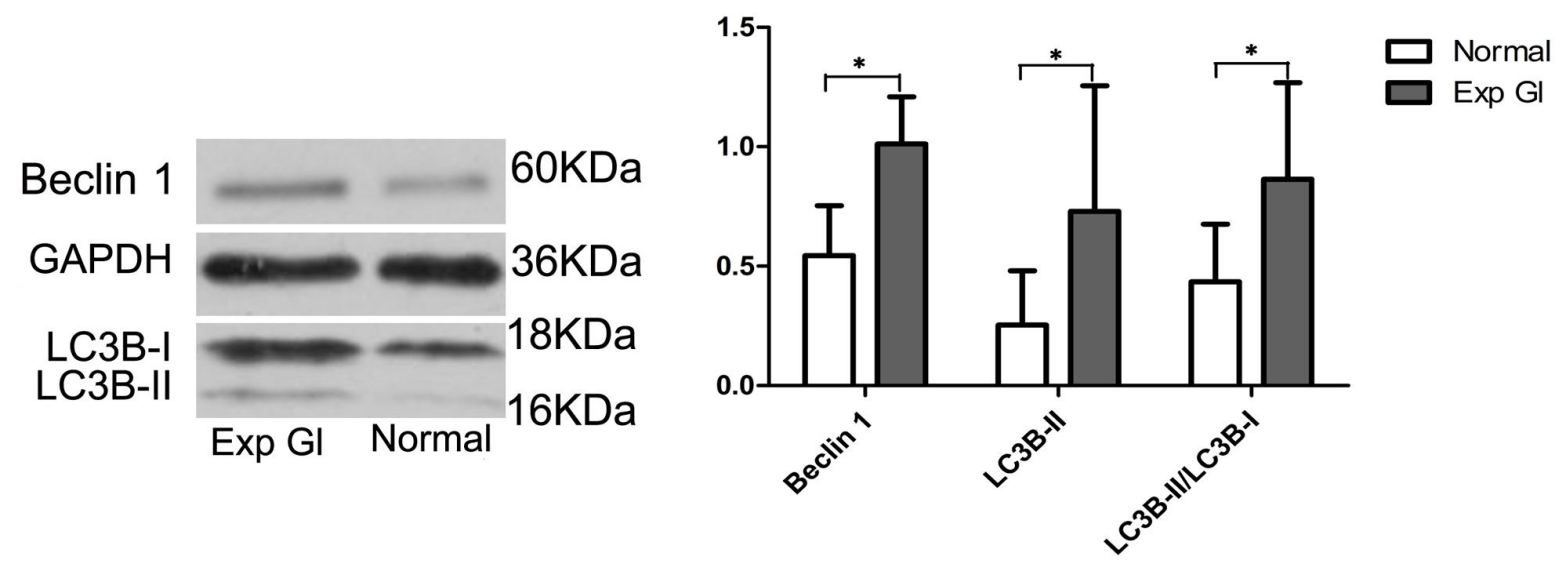
Western blot analyses of the expression of Beclin 1, LC3B-II and LC3B-II/LC3B-I in the normal and exp gl retinas. After normalization to GAPDH, the levels of Beclin 1, LC3B-II and LC3B-II/LC3B-I in the glaucomatous retinas significantly increased compared with the normal retinas (*P<0.05).

To monitor the autophagic process, we first analyzed the autophagic marker proteins Beclin 1 and microtubule-associated protein 1 light chain 3 (LC3) through Western blot analyses. Compared with normal retinas, the levels of Beclin 1 and LC3B-II and the ratio of LC3B-II to LC3B-I were significantly increased in the glaucomatous retinas (Figure 3) (*P*<0.05), indicating the upregulation of autophagic marker protein expression levels in the glaucomatous retinas. 

### Distribution of LC3B in the retina after chronic IOP elevation

**Figure 4:**
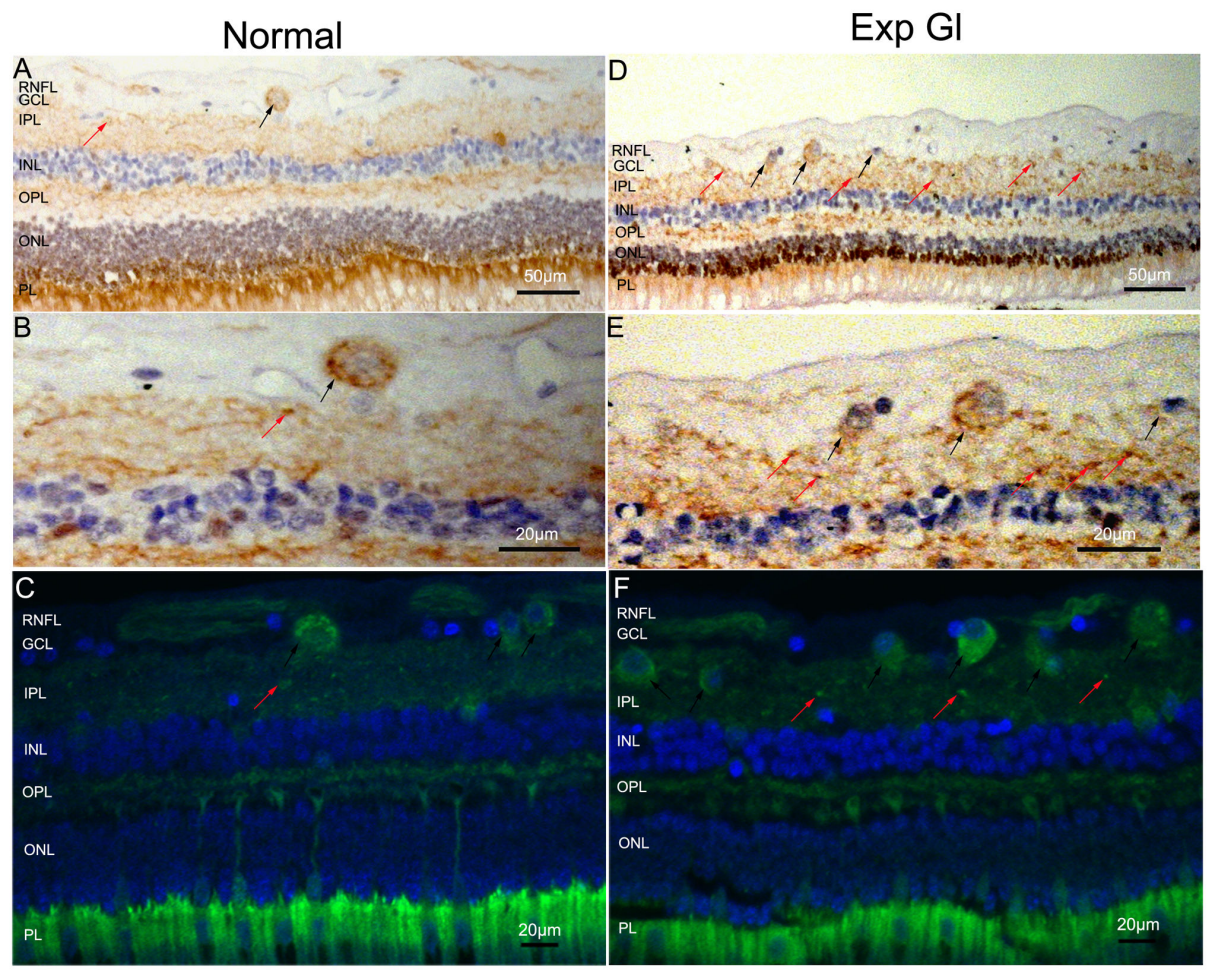
Immunolocalization analysis of LC3B in the normal and exp gl retinas. The nucleus (blue) was immunolabeled with hematoxylin for immunoperoxidase labeling (A, B, D and E) and with DAPI for immunofluorescent analysis (C and F). LC3B immunoreactivity was observed as clusters of small, intensely stained granules, which were brown in A, B, D and E and green in C and F. A, C, D and F. Strong increases in LC3B expression were observed in the GCL (black arrows) and IPL (red arrows) in the glaucomatous retinas compared with the normal retinas. B, C, E and F. With higher magnification, a markedly increased number of positive granules are visible in the cytosol of the RGC in the GCL (black arrows) and in the dendrites of the RGC in the IPL (red arrows) in the glaucomatous retinas compared with the normal retinas. (A, D scale bar=50 μm; B, C, E and F scale bar=20 μm). .

To evaluate the cellular localization of LC3B in the retina, we performed immunohistochemical analyses against LC3B. This analysis demonstrated that in normal retinas, LC3 labeling was faint and heterogeneously distributed in the cytosol of RGC in GCL and in the dendrites of RGC in IPL (Figure 4). However, in the glaucomatous retinas, the number of LC3B-positive RGC in GCL and LC3B-positive granules in IPL significantly increased, and the IPL and most of the RGC displayed stronger labeling of LC3B compared with normal retinas (Figure 4). This result indicates that LC3B immunoreactivity increased in the glaucomatous eyes.

### Ultrastructural features of autophagy after chronic IOP elevation

**Figure 5:**
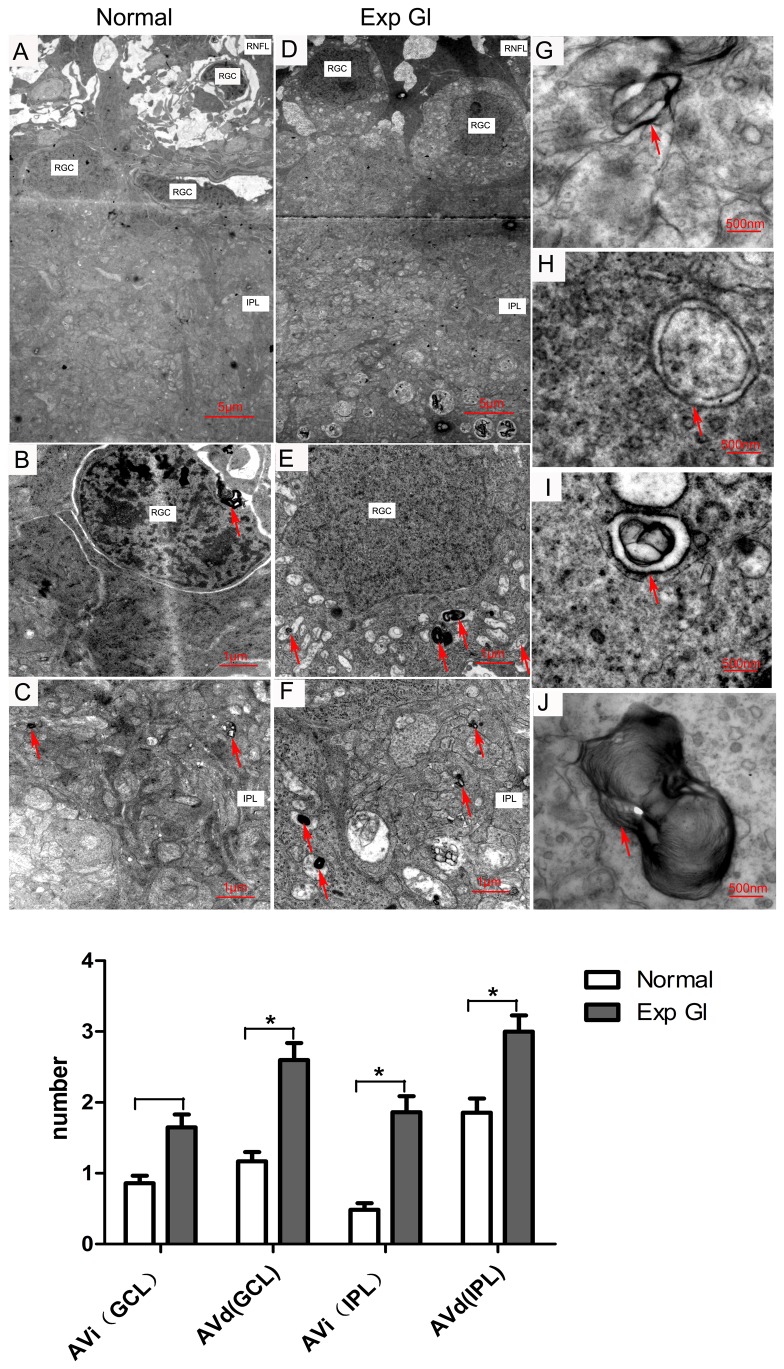
AV formation in transmission electron micrographs of retinal sections. A–C. In the normal retinas, the AVs (red arrows; mostly AVd) were occasionally visible in the cytoplasm of RGC and in the IPL. D-F. In the glaucomatous retinas, the AVs (red arrows; mostly AVd) significantly increased and were heterogeneously distributed in the cytoplasm of RGC and in the IPL (*P<0.05). G and H. AVi, the double-membrane bound vacuoles containing morphologically intact mitochondria (red arrow in G) and cytoplasm (red arrow in H). I and J. AVd, double- or multiple-membrane vacuoles containing partially disintegrated contents (red arrow in I) and whorls of membranous material (red arrow in J). (A, D scale bar=5 μm; B, C, E and F scale bar=1 μm; G-J scale bar=500 nm). Below: Quantification of AVs in the normal and exp gl retinas (*P <0.05). The number of AVi and AVd profiles was counted under the microscope at 9,700× from 25 non-repeating micrographs for each sample.

To identify autophagy formation, which was classified as AVi and AVd at the ultrastructural level, we monitored the retinal sections with TEM. Closed double-membrane-bound vacuoles containing morphologically intact cytoplasm or organelles were identified as AVi, whereas double- and multiple-membrane vacuoles containing partially disintegrated or electron-dense contents or whorls of membranous material were identified as AVd (Figure 5) [9].

Because the immunohistochemical analysis suggested that LC3B immunoreactivity increased in the GCL and IPL in the glaucomatous retinas, we further compared the number of AVs in the GCL and IPL in both normal and glaucomatous retinas. The average number of AVs in the GCL and IPL of the glaucomatous retinas increased significantly compared with those of the normal retinas (Figure 5; GCL: 4.12/unit area versus 2.05/unit area, *P*<0.05; IPL: 5.13/unit area versus 2.33/unit area, *P*<0.05). In the GCL, however, there was no significant difference in the AVi between glaucomatous and normal retinas (1.59/unit area versus 0.87/unit area, *P*>0.05), whereas a significant difference was observed in the AVd (Figure 5; 2.66/unit area versus 1.18/unit area, *P*<0.05). In the IPL, there was a significant difference in both the AVi and AVd between glaucomatous and normal retinas (Figure 5; AVi: 1.88/unit area versus 0.48/unit area, *P* <0.05; AVd: 3.25/unit area versus 1.85/unit area, *P*<0.05). The AVs were heterogeneously distributed in the GCL and IPL, and the high density of AVs was occasionally visibly stacked in the cytoplasm or dendrites of RGCs (Figure 5). AVi and AVd were occasionally observed in the RNFL.

### Change of lysosomal activity and autophagosomal fusion after chronic IOP elevation

**Figure 6:**
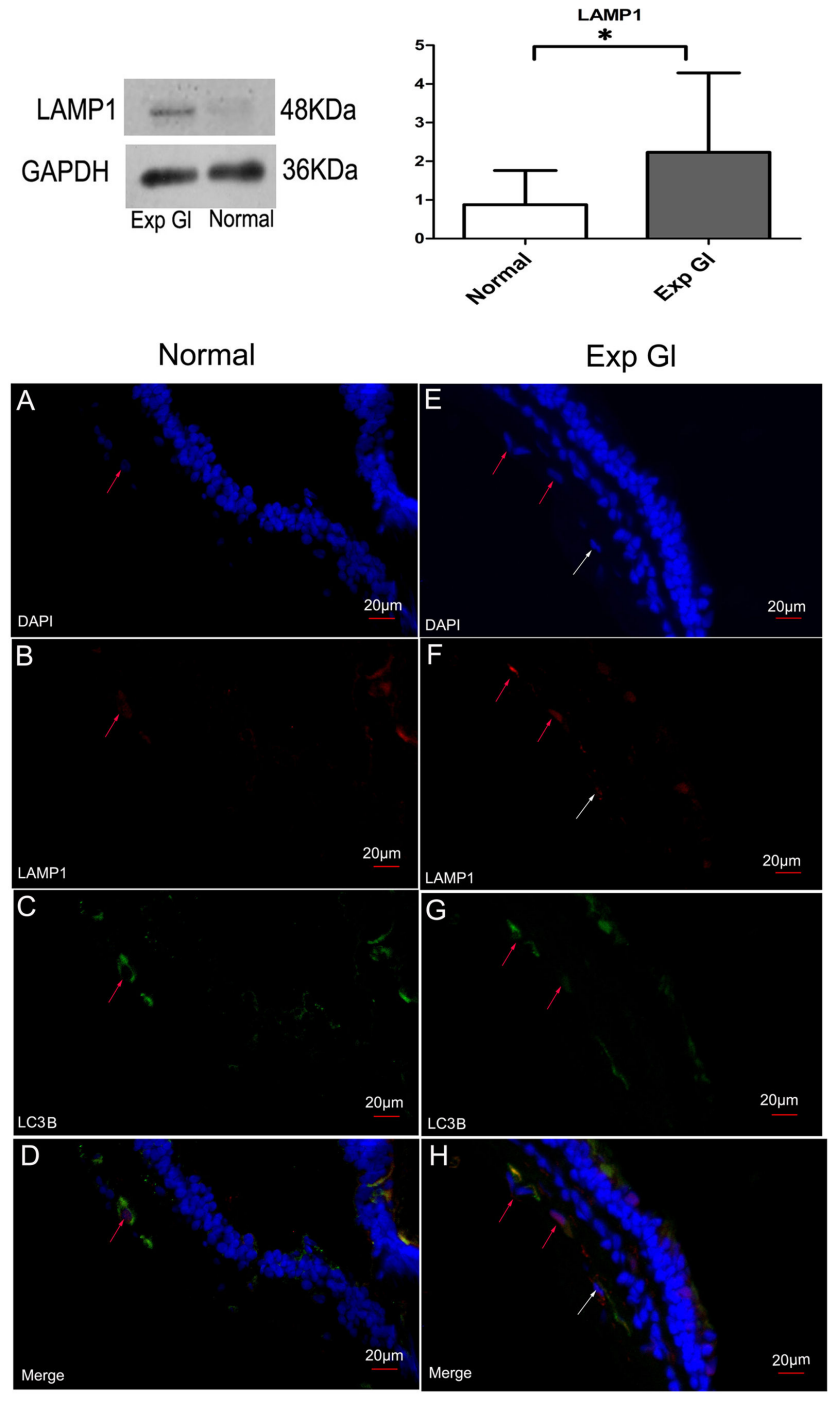
Above: Western blot analysis of LAMP1. After normalization to GAPDH, LAMP1 levels in the glaucomatous retinas increased significantly compared with the normal retinas (*P <0.05). Below: The double immunolabeling for LC3B and LAMP1. The nucleus (blue) was immunolabeled with DAPI, and all retinal sections were stained with the same concentration of antibody against LC3B (green) and LAMP1 (red). In A-D, both LC3B and LAMP1-positive RGC with positive granules in the cytoplasm (red arrow) were occasionally observed in the normal retinas. E-H, frequent LC3B-positive RGC with increased LAMP1-positive granules in the cytoplasm (red arrows) were observed in the glaucomatous retinas, indicating that the fusion of autophagosomes with lysosomes increased in the neurons. Furthermore, LAMP1-positive RGC without LC3-positive granules was visible in the cytoplasm of the glaucomatous retinas (white arrows). Scale bar=20 μm.

To estimate whether AVi accumulation, which was observed in the TEM analysis, was due to an increase in the autophagic flux or a blockage in autophagic degradation, we analyzed the expression of LAMP1 via Western blot analysis and performed double immunolabeling against LC3B and LAMP1. LAMP1 levels increased significantly in the glaucomatous retinas compared with the normal retinas (Figure 6; *P*<0.05); this observation indicates an upregulation of lysosomal activity in the glaucomatous retinas. Furthermore, RGCs positive for LC3B or LAMP1 labeling were frequently observed in the GCL of the glaucomatous retinas, and most LC3B-positive RGCs also displayed increased LAMP1 labeling in the RGC cytoplasm. Such cases were only occasionally observed in the GCL of the normal retinas (Figure 6). This distinction underlines the increases in the fusion of autophagosomes with lysosomes in the RGC of glaucomatous retinas.

### Detection of apoptosis after chronic IOP elevation

**Figure 7:**
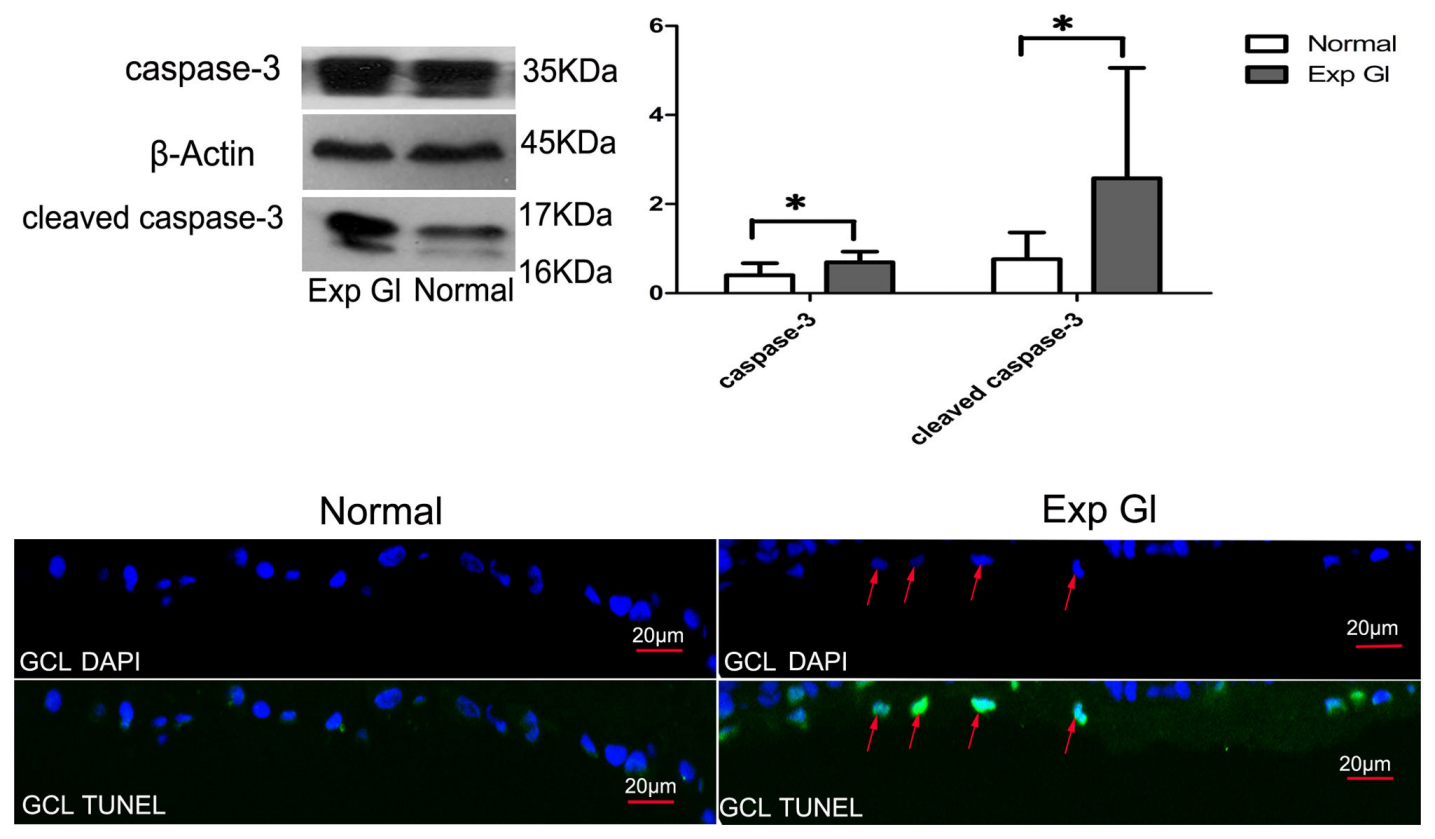
Above: Western blot analyses for caspase-3 and cleaved caspase-3. After normalization to β-Actin, the levels of caspase-3 and cleaved caspase-3 in the glaucomatous retina increased significantly compared with the normal retinas (*P <0.05). Below: TUNEL staining of the normal and exp gl retinas. Few TUNEL-positive RGCs were observed in the normal retina, whereas a larger number were observed in the glaucomatous retina in the GCL (red arrow). Scale bar=20 μm.

To estimate whether apoptosis was present in the glaucomatous eyes of the rhesus monkeys, we analyzed the expression of caspase-3 as well as its activated form, cleaved caspase-3, via Western blot analysis and detected apoptotic cells through TUNEL staining analysis. Our results indicated a significant increase in caspase-3 and cleaved caspase-3 expression levels (*P*<0.05) as well as in the number of apoptotic RGCs in the glaucomatous retinas compared with the normal retinas (Figure 7). This result indicates that the chronic IOP elevation induced apoptosis in the treated eyes.

## Discussion

In the present study, we demonstrated that autophagy, aside from apoptosis alone, was also activated in the RGC of a rhesus monkey model of chronic ocular hypertension using different methods suitable for studying autophagic flux in vivo. The increased autophagic flux in the glaucomatous eyes was demonstrated by the enhancement of LC3B-II, LC3B-II/LC3B-I and Beclin 1 together with the accumulation of AVs and the upregulation of lysosomal activity and autophagolysosomal fusion in the glaucomatous retinas. Concurrently, apoptosis was induced in glaucomatous eyes as noted by an increase in caspase-3 and cleaved caspase-3 expression levels and TUNEL-positive RGC numbers in the glaucomatous retinas. Our results are consistent with the conclusions of previous research using this model in rodents [8].

Regardless of the wide range of IOP values and durations between the rhesus monkeys, the cup/disc ratio enlargement and quantitative RNFL thickness defects have provided reliable evidence for RGC deficits in the chronic glaucoma model [1,26–29]. Progressive cupping is characteristic of glaucomatous optic nerve damage [27,28], and the quantitative estimation of RNFL thickness is one of the most sensitive parameters for detecting the structural damage of glaucoma in primates [24,30]. The extent and pattern of RGC deficits have been reported to mimic those of humans in the experimental glaucoma model of the rhesus monkey [27,28], and apoptosis has been suggested as the final pathway for RGC loss in chronic glaucoma for several decades [1,31]. We demonstrated that caspase-dependent apoptosis is triggered in the glaucomatous retinas with the elevated caspase-3 and cleaved caspase-3 levels. In recent years, autophagy has also been shown to play an important role in RGC loss [5–8]. Given that autophagy is a highly dynamic process that is regulated at multiple steps, we demonstrated an increase in autophagic flux in the glaucomatous retina using comprehensive methods.

The number of AVs increased in the RGC of the glaucomatous eyes in our study. During the autophagic process, cytosolic LC3-I is cleaved and conjugated with phosphatidylethanolamine (PE). At this point, the cell is converted to the autophagic form, LC3-II [32,33]. LC3-II is the only known Atg protein that remains in the membrane of the mature autophagosomes. The amount of LC3-II typically correlates well with the number of autophagosomes, which is classified as AVi [34]. Consistent with this notion, the apparently elevated LC3-II levels by immunoblotting and increased LC3-positive vesicles by immunolabeling in RGC in the glaucomatous eyes suggested AV abundance in our model. Furthermore, the quantitative estimation of AV formation provided direct evidence for the accumulation of AVs in the glaucomatous eyes.

Lysosomal activity and autophagosomal fusion increased in the glaucomatous eyes. An accumulation of AVs may represent either the induction of autophagy or, alternatively, the suppression of steps in the autophagy pathway downstream of autophagosome formation by blocking autophagolysosomal fusion or reducing lysosomal activity [9,15,23]. Many studies have observed AV abundance in certain neurodegenerative diseases; however, this abundance was finally proven due to autophagy dysregulation with lysosomal malfunction [12,35,36]. LAMP1, which is absent in AVi but abundant in AVd [13,14], may be involved in the interaction and fusion of the lysosomes with AVi, thus regulating the autophagic flux [15]. Some researchers have observed a decrease in the co-localization of LAMP1 and LC3 in neurodegenerative diseases with lysosomal malfunction [36]. We observed elevated LAMP1 levels and increased co-localization of LC3-positive vesicles and LAMP1-labeled vesicles in the RGC of the glaucomatous retinas, suggesting that the lysosomal activity and fusion of autophagosomes with lysosomes was increased in the glaucomatous retinas. In addition, the increased number of AVd observed in the TEM analysis indicated that the AVi fused with primary lysosomes. This observation demonstrated that AV accumulation was due to an increased autophagic flux instead of a blockage of autophagic degradation, which is consistent with the results of a previous study on acute intraocular pressure elevation [7]. Moreover, a blockage of autophagic degradation is typically associated with an increase in the number of AVi without a change in the number of AVd [23]. The significant increase in the numbers of both AVi and AVd in the RGC among the glaucomatous retinas suggested increased AV synthesis in the glaucomatous eye.

LC3-II turnover increased in the glaucomatous eyes. The measurement of LC3-II presented a similar concern as the accumulation of AVs [23]. A simple elevation of LC3-II levels is insufficient to indicate an autophagic flux. The increased ratio between LC3-II and LC3-I in the glaucomatous retinas of our model indicated the increased rate of LC3-II turnover, which is an increased rate of LC3-II degradation in autolysosomes [32] that provides a reliable indicator of increased autophagic flux in glaucomatous eyes.

Autophagy induction in mammalian cells is mainly dependent upon the activation of the class III phosphatidylinositol kinase (PI3K) or threonine kinase (TOR) pathways [37]. Beclin 1 is a cytosolic protein that is part of the Class III PI3K machinery that participates in autophagosome formation [38,39]. Many studies have demonstrated a critical role for Beclin 1 in autophagy induction [40,41]. Increased Beclin 1 expression was also observed in the glaucomatous retinas in our study, demonstrating that the upregulation of Beclin 1 occurred in response to the glaucomatous RGC damage, whereas at least the class III PI3K pathway was activated to increase the autophagic flux of glaucomatous eyes. Interestingly, some studies have reported that Beclin 1 is downregulated through a calpain-mediated cleavage pathway; however, calpain has been suggested to be required for autophagy induction in mammalian cells [40,42]. This potential role may lead us to draw the paradoxical conclusion that Beclin 1 is reduced when autophagy is induced. The relationship between Beclin 1 and calpain in the autophagic process still remains to be investigated. Nonetheless, taken together, our results demonstrated that there was increased autophagic flux in the glaucomatous retinas.

In addition to its role as a regulator of autophagy, Beclin 1 was involved in the intriguing link between the pathways controlling autophagy and apoptosis [43]. Studies have shown that an increase in Beclin 1 expression can co-localize with caspase-3 activation [44,45], whereas the inhibition of autophagy reduces caspase-3 activation in some ischemic diseases of the CNS [44,46]. Autophagy may trigger apoptosis in neuronal death [19]. In the glaucomatous retinas of our model, we demonstrated that autophagy is induced with increased Beclin 1 levels, whereas apoptosis is triggered by increased caspase-3 and cleaved caspase-3 levels simultaneously, which is in accordance with previous work [7,8]. These results indicate that the activation of autophagic and apoptotic mechanisms may occur simultaneously in glaucomatous retinas. However, whether enhanced autophagy is present as an independent mechanism or as a precursor of apoptosis remains unclear; furthermore, if the latter is true, it remains unknown whether enhanced autophagy exists as a protective mechanism or contributes to neuronal death.

The regulation of autophagy remains largely unknown in neurons. In glaucomatous retinas, AVs mainly accumulate in the dendrites and cytoplasm of RGCs, and only a few AVs can occasionally be found in the axons of RGCs in the RNFL. This conclusion is consistent with those of previous reports on a chronic hypertensive glaucoma rat model and the model of optic nerve axotomy in rats [6,8]. A large number of AVs have been observed to accumulate in the cytosol of neurons in certain CNS diseases when autophagic flux is enhanced [19]. Other reports, however, revealed that efficient autophagosome production is induced in axons under stressful conditions and accumulates in the axon terminals or distal ends due to a blockage of retrograde axonal transportation, which could be induced by sustained IOP elevation [47,48]. Our results and the facts described above suggest that autophagy may be regulated differently in different RGC compartments [8,47]. Nevertheless, a clear mechanism of how activated autophagy occurs and develops remains to be investigated.

In summary, we present reliable evidence for the activation of autophagy, in addition to apoptosis in the retinas of a rhesus monkey model of chronic hypertensive glaucoma. As the monkey model of laser-induced ocular hypertension is considered by many to be the best animal model to study human glaucoma [25,27], our results may predict a new target for intervention other than apoptosis in the treatment of glaucoma. However, because of our limited sample size, these findings should be considered preliminary. Further studies are required to analyze the detailed mechanisms and functions involved in autophagy and glaucoma and to explore therapeutic measures for chronic hypertensive glaucoma.
